# Porcelain: A Fad or a Fact

**Published:** 1903-11

**Authors:** James Ross

**Affiliations:** Fitchburg, Mass.


### PORCELAIN: A FAD OR A FACT.[1]

BY DR. JAMES ROSS, FITCHBURG, MASS.

In asking the question, "Is the so-called porcelain worker of to-day a faddist?" we might answer, that it depends on the spirit in which he has taken up the work. In the March number (this year) of the *Dental Cosmos* is a paper by Dr. C. N. Johnson, of Chicago, on "The Professional Spirit." That paper struck a responsive chord in me, and I wish every man in our profession might read it thoughtfully and profit by its high ethical teaching, and yet what he calls "the bone and sinew" of our professional faith,—a spirit of broad humanitarianism which looks only to the

---

[1] Read before the Massachusetts Dental Society, Boston, Mass., June 3 and 4, 1903.

greatest good for the greatest number, and which rigidly excludes selfishness of motive or narrowness of purpose.

The tendency, perhaps, in all professions to-day, but surely in ours, is to descend to the trade or commercial spirit, and I say *descend* without any disrespect for the tradesman or the man of business. The true professional man gives free to the world the results of many weary days and nights of thought and study. This is what I mean by the *spirit* in which we have entered this field of our work. I am glad to give my testimony that the men I have met as pioneers—leaders—in this work have impressed me as men of honesty and earnestness of purpose. But it is not to the few bright lights, but to the many humble workers, who, like myself, wish to avoid the fads and take up the facts, that I wish to give a few practical rules as a working basis. First, be convinced by what you have seen and heard, that it is a real part of our profession. Find fault as much as you wish with cement joints, imperfect colors, misfits, and the like, but finally make up your mind that it really does preserve teeth and do service in many places better than any other filling we now have. Again, make up your mind whether you have enough of that true professional spirit that will allow you to spend money and time for the good of humanity and your profession, when you might be making money putting in fillings of gold, silver, and cement. But if you belong to that too large class who put gold caps on teeth, from centrals to third molars, simply for the money there is in it, don't for a minute think of porcelain.

So much has been written of late years about the methods of using porcelain for fillings, that it seems almost unnecessary to write a paper on that subject, as it must be only repetition, but still we must repeat, and continue to improve.

If we have really decided to put porcelain alongside of other materials in our practice, the first thing to determine is the method. Experiment as much as you wish, buy as many outfits as you can afford later on, but at first decide on one method, and do some thorough practical work, for be convinced that this is no catephoric painless dentistry fad, but that it has come to stay. Your own first inlays may not.

Now, in a broad way, the two methods are high- and low-fusing porcelain bodies. It is not my intention to criticise or champion either method, as I have seen most excellent work done by both, but my own actual experience has been with high-fusing bodies. The difference between the two methods is that in high fusing you must use platinum 0.001 for the matrix, and in low fusing, gold-foil No. 30 or 40 in some cases. The platinum matrix can be placed directly in the furnace, but the gold must be embedded in powdered asbestos. Do not try to place a porcelain filling where you cannot get a perfect matrix at the start. Do not try pin-head cavities. In other words, there are safe, doubtful, and unsafe cavities for porcelain.

Among the most suitable places are the cervical margins of the labial and buccal surfaces of all the teeth from right bicuspids to left bicuspids, upper and lower, but particularly upper. Also proximate cavities, corners and cross-sections, mesial or compound cavities in bicuspids or first molars, provided there is room. Use no rubber dam, unless to insert completed filling, and even that is not necessary, only permissible. And the reason is that it will fool you on color, and your patient is much more comfortable without it.

Anneal your platinum in the furnace. Do not allow the platinum to double on the edges, but never mind the breaks in the bottom of the matrix. Have the porcelain as dry as possible, and run under tapping. Usually burnish a second time after the first bake. Never take off the matrix until you are sure the filling is finished, as you cannot fuse after that is done. To remove platinum, peal from edges back to centre. Insert with cement, quite soft, being careful of color.

Now let us for a little consider that other important part of our subject, that comes so frequently into our practice after neglect or accident has done its work,—crowns. You will pardon me if I speak forcibly and plainly in advocating porcelain or artistic work as against the indiscriminate use of gold caps. I say that any man who puts a whole gold cap on any of the incisors except in rare cases, does not know his whole business. Then what can be done? Use one of the many styles of porcelain crowns. Mind you, I do not condemn all gold crowns, for there is nothing like them for molars and bicuspids in many cases.

There are two classes of teeth that are extremely bothersome, and I suppose they are the ones that so many of our brethren feel must be capped with gold, as there is nothing else to do with them, even on centrals and laterals. The first of these is the root that is hollowed out so badly by neglect or split from using some style of pin crown. You cannot use a pin crown and expect it to hold in place with cement or gutta-percha, but it can be built in with amalgam. The second is the accidentally broken tooth, but with the pulp still protected and healthy.

Of course, there have been several kinds of crowns on the market for years, to be used with the post built in, but to those who have a furnace for porcelain work the variety is largely increased. The first and best of that kind is the jacket crown, advocated so strongly and used with great success for years by my instructor in this work, Dr. W. A. Capon, of Philadelphia. With the jacket crown you get the same principle as the gold cap, which I think is the strongest principle, and for bicuspid teeth, when you have a fastidious and æsthetically inclined patient, they are much more acceptable.

Then there is the banded pin crown (similar to Richmond) and the tube crown, which is strong, quickly made, and inexpensive, and a large number of variations for the ingenious worker.

In closing, I again wish to refer to the professional spirit, which is broadening in its tendencies and takes a man out of the narrow rut of self-conceit, and if I have in the least added to the inspiration to make our profession a constructive one in the line of beauty, as it is in nature, then will I count this time well spent.

